# Inhibition of NLRP3 inflammasome in tumor microenvironment leads to suppression of metastatic potential of cancer cells

**DOI:** 10.1038/s41598-019-48794-x

**Published:** 2019-08-22

**Authors:** Hye Eun Lee, Jin Young Lee, Gabsik Yang, Han Chang Kang, Yong-Yeon Cho, Hye Suk Lee, Joo Young Lee

**Affiliations:** BK21 PLUS Team, College of Pharmacy, The Catholic University of Korea, Bucheon, 14662 Republic of Korea

**Keywords:** Inflammasome, Pattern recognition receptors

## Abstract

Tumor microenvironment favors tumor cells to promote their growth and metastasis such as migration, invasion, and angiogenesis. IL-1β, one of the inflammatory cytokines released from myeloid cells in tumor microenvironment, plays an important role in development and progress of tumor. The activation of inflammasome is a critical step to secrete mature IL-1β through stepwise reactions to activate capspase-1. Therefore, we investigated whether the inhibition of NACHT, LRR and PYD domains-containing protein 3 (NLRP3) inflammasome in macrophages regulated the metastatic potential of tumor cells. NLRP3 inflammasome was activated by ATP in bone marrow-derived primary mouse macrophages. The metastatic potential of mouse melanoma cell line (B16F10) was determined by migration and invasion assays with transwell system. ATP-treated wild-type macrophages increased the migration and invasion of melanoma cells. However, NLRP3- or caspase-1-knockout macrophages exhibited greatly diminished ability to promote the migration and invasion of melanoma cells. In addition, treatment with celastrol, an inhibitor of NLRP3 inflammasome, reduced the potency of macrophages to stimulate migration and invasion of melanoma cells. The results demonstrate that inhibition of the NLRP3 inflammasome in macrophages by genetic deficiency or a pharmacological inhibitor is linked to suppression of the metastatic potential of tumor cells. The results would provide a novel anti-cancer strategy to modulate tumor microenvironment by suppressing NLRP3 inflammasome and consequently reducing IL-1β production.

## Introduction

Tumor microenvironment favors tumor cells to promote tumor growth, migration, invasion, and angiogenesis. One of the main features of tumor microenvironment is inflammation accompanied with the activation of immune cells and the production of pro-inflammatory cytokines^1,2^. IL-1β is one of the critical cytokines to promote tumor growth and metastasis by acting on its receptor, IL-1R, and to culminate in the secondary activation of immune cells and the expression of immune cytokines^3^. IL-1β requires maturation process of cleavage from pro-IL-1β transcribed via inflammatory signals and is secreted to extracellular matrix to act on its receptor on cellular membrane. Caspase-1 is the intracellular enzyme responsible for cleavage of pro-IL-1β to IL-1β. The activation of caspase-1 occurs upon the activation of inflammasomes which induces the degradation of pro-caspase-1 (pro-form) to caspase-1 (active form)^4^. Therefore, the regulation of inflammasomes by pharmacological inhibitors is considered as an efficient strategy to reduce IL-1β levels in various inflammatory and immune diseases^5,6^.

NACHT, LRR and PYD domains-containing protein 3 (NLRP3) inflammasome is a protein complex composing of the NLRP3, an adaptor protein, and pro-caspase-1. NLRP3 inflammasome is activated by recognizing a diverse set of inflammation-inducing stimuli that include pathogen-associated molecular patterns (PAMPs) and damage-associated molecular patterns (DAMPs)^7^. The activation of the NLRP3 inflammasome by the DAMPs leads to the activation of intracellular signaling pathways such as increase in potassium efflux, production of reactive oxygen species, and lysosomal damage^8^. In particular, adenosine triphosphate (ATP) is the representative DAMPs that are endogenous substances derived from the host to activate NLRP3 inflammasome^9^. ATP is released from damaged and dying cells to extracellular matrix, therefore, accumulated in high levels in inflamed site and tumor microenvironment^10^. These suggest that ATP accumulated in tumor microenvironment would activate NLRP3 inflammasome resulting in the increased secretion of IL-1β from immune cells.

In this study, we investigated whether the inhibition of NLRP3 inflammasome in macrophages resulted in the regulation of metastatic potential of cancer cells. The results would provide an effective strategy to prevent tumor progression by modulating cytokine expression in tumor microenvironment. The suppression of NLRP3 inflammasome in immune cells could be a novel strategy for anti-cancer therapy by reducing IL-1β secretion.

## Results

### Deficiency of NLRP3 inflammasome in macrophages results in reduction of metastatic potential of cancer cells

**Figure 1:**
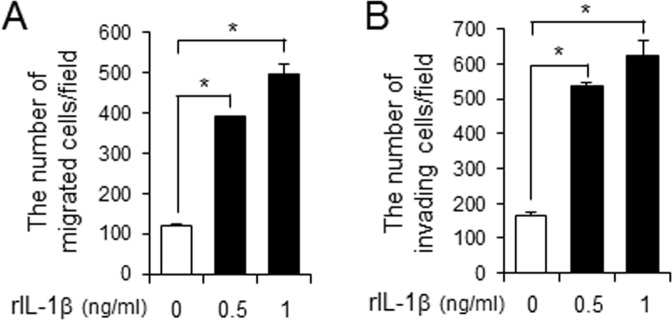
IL-1β increases the metastatic potential of melanoma cancer cells. (**A**,**B**) B16F10 cells were plated in upper chamber of transwell. Serum-free medium containing recombinant IL-1β (0.5, 1 ng/ml) was placed in the bottom chamber. After 24 hr, the migration or invasion of B16F10 cells was determined. Values are means ± SEM (n = 3). *p < 0.05.

Since IL-1β is the major cytokine secreted upon NLRP3 inflammasome activation in macrophages, we investigated whether IL-1β would increase the metastatic potential of cancer cells. Treatment of recombinant IL-1β increased the number of migrated melanoma cancer cells (B16F10) in transwell system (Fig. 1A). In addition, recombinant IL-1β increased the number of invading melanoma cancer cells (Fig. 1B). These results demonstrate that IL-1β can enhance the metastatic potential of cancer cells.

**Figure 2:**
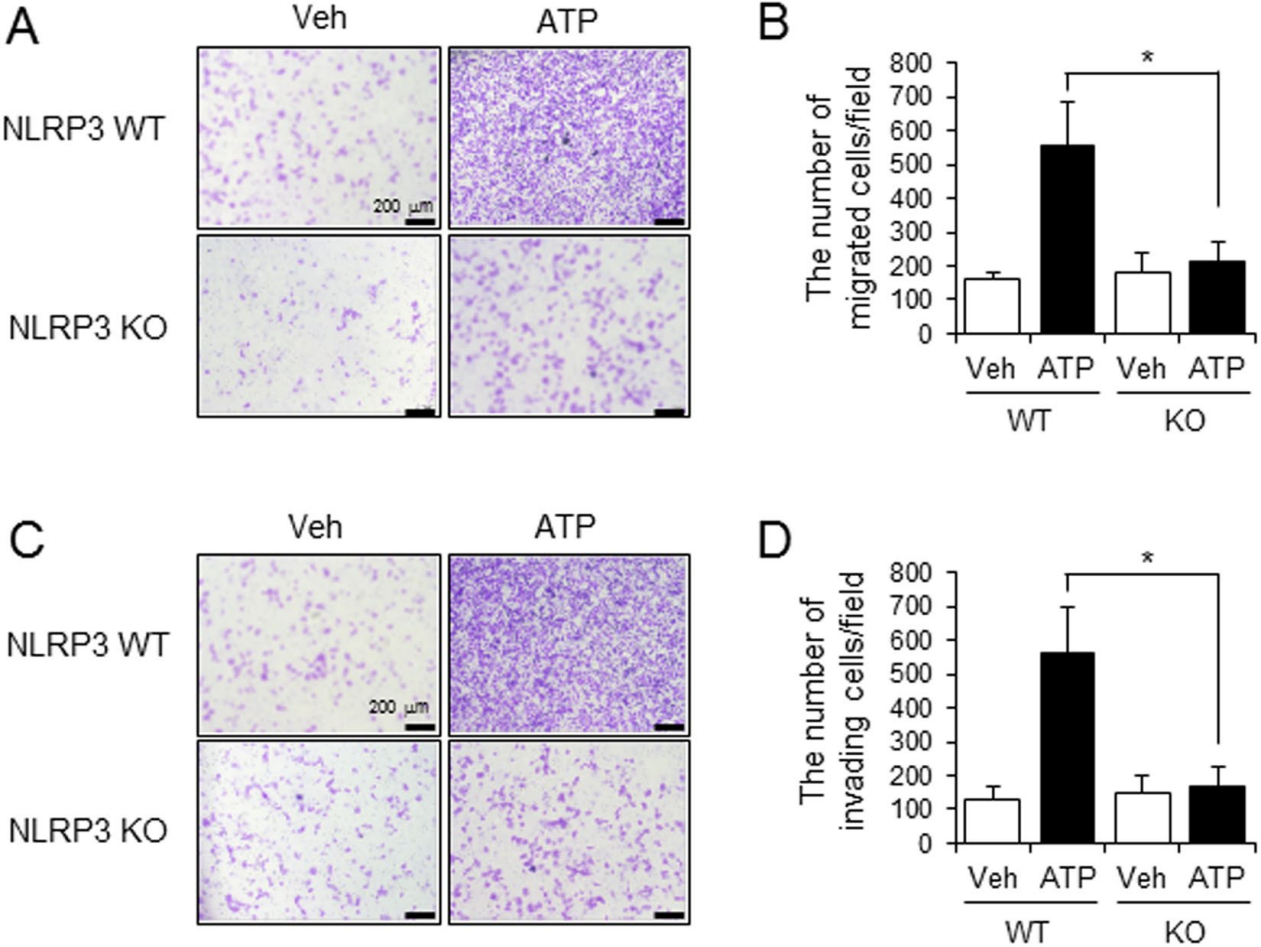
NLRP3-deficient macrophages failed to increase the metastatic potential of melanoma cancer cells. Primary mouse macrophages derived from wild-type (WT) or NLRP3 knockout (KO) mice were primed with LPS (100 ng/ml) for 4 hr. The cells were stimulated with ATP (5 mM) for 1 hr. Conditioned culture supernatants were collected and added to the bottom chambers of transwells which had B16F10 cells plated in the upper chamber. After 24 hr, cell migration and invasion were determined. (**A**) Images of B16F10 cell migration. (**B**) The number of migrating B16F10 cells was counted in the microscopic field. (**C**) Images of B16F10 cell invasion. (**D**) The number of invading B16F10 cells was counted in the microscopic field. Values are means ± SEM (n = 5) for B and D. *p < 0.05.

We next investigated whether the activation of NLRP3 inflammasome in immune cells in tumor microenvironment contributed to promoting metastatic potential of cancer cells. After primary mouse macrophages derived from wild-type (WT) or NLRP3-knockout (KO) mice were stimulated with ATP, an NLRP3 inflammasome activator, conditioned media was collected. The media was added to the lower chamber of transwell and the migration of cancer cells plated on the upper chamber of transwell was measured. Conditioned media from ATP-stimulated WT macrophages increased the migration of melanoma cancer cells (B16F10) as compared with vehicle-treated WT macrophages (WT, vehicle vs WT, ATP: 158.8 ± 20.33 vs 553.8 ± 130.09) (Fig. 2A,B). In contrast, the ability to induce the cancer cell migration was dramatically reduced when conditioned media from ATP-stimulated NLRP3 KO macrophages was added (WT, ATP vs KO, ATP: 553.8 ± 130.09 vs 214.6 ± 59.07) (Fig. 2A,B).

We further examined whether the activation of NLRP3 inflammasome in macrophages enhanced the invasive activity of cancer cells. Conditioned media obtained from ATP-stimulated WT macrophages increased the invasive potential of melanoma cancer cells (B16F10) (WT, vehicle vs WT, ATP: 132 ± 38.05 vs 564.6 ± 133.52) (Fig. 2C,D) while conditioned media collected from NLRP3 KO macrophages stimulated with ATP failed to enhance invasion of cancer cells (WT, ATP vs KO, ATP: 564.6 ± 133.52 vs 170.6 ± 55.48) (Fig. 2C,D).

**Figure 3:**
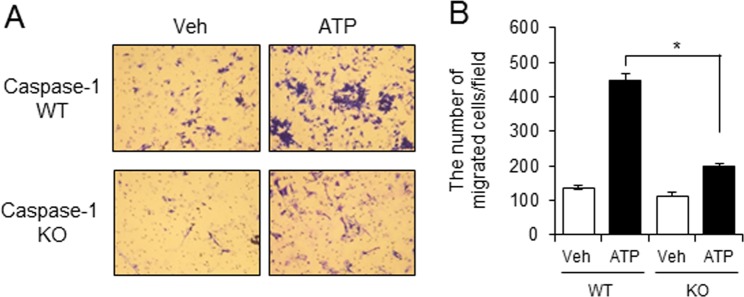
Caspase-1-deficient macrophages have greatly reduced capacity to stimulate migration of melanoma cancer cells. Primary mouse macrophages derived from wild-type (WT) or caspase-1 knockout (KO) mice were primed with LPS (100 ng/ml) for 4 hr. The cells were stimulated with ATP (5 mM) for 1 hr. Conditioned culture supernatants were collected and added to the bottom chambers of transwells which had B16F10 cells plated in the upper chamber. After 24 hr, cell migration was determined. (**A**) Images of B16F10 cell migration. (**B**) The number of migrating B16F10 cells was counted in the microscopic field. Values are means ± SEM (n = 3). *p < 0.05.

We confirmed the involvement of NLRP3 inflammasome using caspase-1 deficient macrophages. Conditioned media from ATP-stimulated WT macrophages increased the migration of melanoma cancer cells (B16F10) (Fig. 3) while the migration of melanoma cancer cells was decreased when conditioned media from caspase-1 KO macrophages stimulated with ATP was added (Fig. 3).

**Figure 4:**
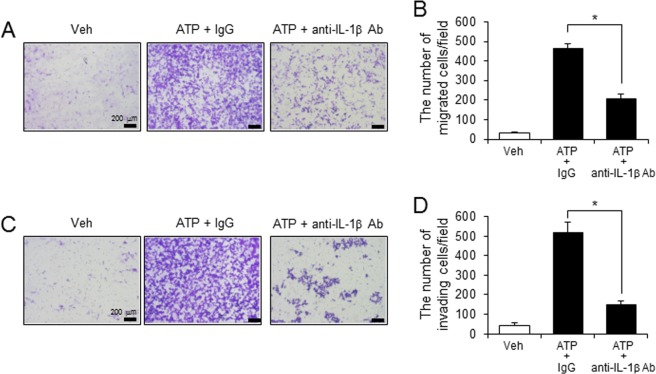
Tumor-promoting activity of macrophages is mediated through IL-1β. Primary mouse macrophages primed by LPS (100 ng/ml) were stimulated with ATP (5 mM) for 1 hr in the presence of isotype control IgG or anti-IL-1β antibody (10 μg/ml). Conditioned culture supernatants were collected and added to the bottom chambers of transwells which had B16F10 cells plated in the upper chamber. After 24 hr, cell migration and invasion were determined. (**A**) Images of B16F10 cell migration. (**B**) The number of migrating B16F10 cells was counted in the microscopic field. (**C**) Images of B16F10 cell invasion. (**D**) The number of invading B16F10 cells was counted in the microscopic field. Values are means ± SEM (n = 5). *p < 0.05.

To investigate whether tumor-promoting activity induced by cell supernatants of ATP-stimulated macrophages was mediated by IL-1β, the macrophages were treated with anti-IL-1β antibody after stimulation with ATP. Anti-IL-1β antibody prevented the migration and invasion of melanoma cancer cells (B16F10) induced by cell supernatants of ATP-stimulated macrophages (Fig. 4).

The results show that the deficiency of NLRP3 inflammasome components in macrophages leads to suppression of metastatic potential of cancer cells by reducing migration and invasion. These suggest that the activation of NLRP3 inflammasome in macrophages by ATP of which level is high in tumor microenvironment, results in the increased production of IL-1β and the promotion of tumor cell metastasis.

### Celastrol, an inhibitor of NLRP3 inflammasome, reduces macrophage ability to stimulate migration and invasion of cancer cells

**Figure 5:**
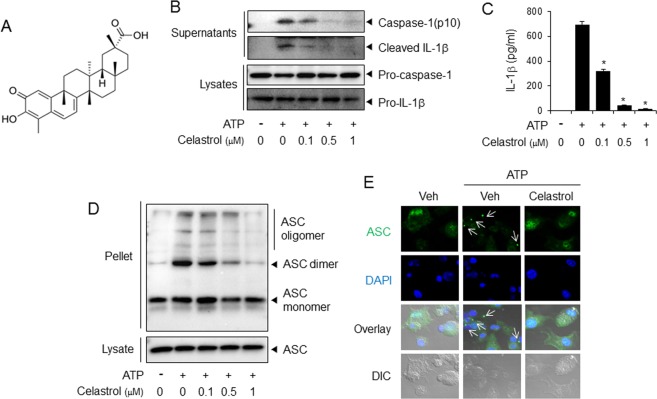
Celastrol inhibits ATP-induced activation of NLRP3 inflammasome in primary macrophages. (**A**) Chemical structure of celastrol. (**B**–**E**) Primary mouse macrophages were primed with LPS (100 ng/ml) for 4 hr. The cells were treated with celastrol for 1 hr and then stimulated with ATP (5 mM) for (**B**,**D**,**E**) 1 hr or (**C**) 2 hr. (**B**) The cell culture supernatants and cell lysates were immunoblotted for pro-caspase-1, caspase-1(p10), pro-IL-1β, and IL-1β. (**C**) The cell culture supernatants were analyzed for secreted IL-1β using ELISA. The values represent the means ± SEM (n = 3). *Significantly different from ATP alone, *p* < 0.05. (**D**) Cell lysates were processed for immunoblotting of ASC. (**E**) The cells were stained for ASC (green). The nuclei were stained with 4′,6-diamidino-2-phenylindole (DAPI; blue). The arrows indicate ASC speckles. The data are representative of three independent experiments. For immunoblotting results, the cropped blots from full length gels were presented. DIC, differential interference contrast.

Next, we hypothesized that the pharmacological inhibitors of NLRP3 inflammasome would suppress metastatic potential of cancer cells. We found that celastrol (Fig. 5A) was effective to block activation of NLRP3 inflammasome in macrophages (Fig. 5). Celastrol blocked ATP-induced degradation of pro-caspase-1 to caspase-1(p10) and cleavage of pro-IL-1β to mature IL-1β in macrophages as determined by immunoblotting assay (Fig. 5B). Celastrol inhibited ATP-induced secretion of mature IL-1β as determined by ELISA of culture media (Fig. 5C). Oligomerization of apoptosis-associated speck-like protein containing a CARD (ASC) is another hallmark of NLRP3 inflammasome activation. Celastrol blocked ATP-induced ASC oligomerization in macrophages (Fig. 5D). Confocal microscopy analysis showed that celastrol reduced ASC speckle formation induced by ATP (Fig. 5E). These results show that celastrol is effective to suppress ATP-induced activation of NLRP3 inflammasome in macrophages. In contrast, celastrol did not suppress the activation of other inflammasomes such as AIM2 (absent in melanoma 2) and NLRC4 (also known as IPAF) since celastrol did not block the production of caspase-1(p10) and IL-1β induced by poly dA:dT (an AIM2 activator) or flagellin (an NLRC4 activator) in primary mouse macrophages (Supplemental Fig. 1).

**Figure 6:**
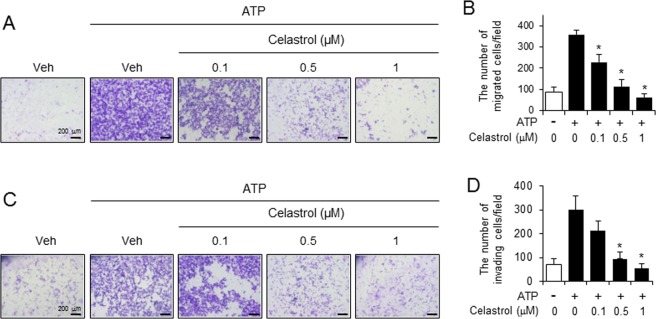
Celastrol treatment reduces the potency of macrophages to stimulate the metastatic potential of melanoma cancer cells. LPS-primed primary mouse macrophages were stimulated with ATP (5 mM) for 1 hr in the presence or absence of celastrol. Conditioned culture supernatants were collected and added to the bottom chambers of transwells which had B16F10 cells plated in the upper chamber. After 24 hr, cell migration and invasion were determined. (**A**) Images of B16F10 cell migration. (**B**) The number of migrating B16F10 cells was counted in the microscopic field. (**C**) Images of B16F10 cell invasion. (**D**) The number of invading B16F10 cells was counted in the microscopic field. Values are means ± SEM (n = 5) for B and D. *Significantly different from ATP alone, *p* < 0.05.

We investigated whether the suppression of NLRP3 inflammasome activation by celastrol in macrophages contributed to the blockade of migration and invasion of cancer cells. Celastrol was treated to the macrophages which were stimulated with ATP and the conditioned media was collected to be added to lower chamber of transwell. The migration and invasion of melanoma cancer cells (B16F10) seeded on upper chamber were determined. Conditioned media derived from ATP-stimulated macrophages induced the migration of melanoma cancer cells (B16F10), while conditioned media obtained from ATP-stimulated macrophages in the presence of celastrol failed to induce cancer cell migration (ATP vs ATP ± celastrol 1 μM: 359 ± 22.04 vs 63.4 ± 16.82) (Fig. 6A,B). Furthermore, celastrol treatment to ATP-stimulated macrophages resulted in the decrease of invasive properties of melanoma cancer cells (ATP vs ATP ± celastrol 1 μM: 301 ± 58.37 vs 54.2 ± 20.36) (Fig. 6C,D).

These results indicate that celastrol treatment to macrophages suppresses the stimulatory effects of macrophages on cancer migration and invasion, possibly mediated through the inhibition of NLRP3 inflammasome activation and the consequent reduction of IL-1β secretion.

### Celastrol suppressed the potassium efflux induced by ATP and nigericin in macrophages

We further investigated the mechanism as to how celastrol inhibited ATP-induced NLRP3 inflammasome. Cellular mechanism by which ATP activates NLRP3 inflammasome includes the activation of the purinergic P2X7 receptor and the recruitment of pannexin-1 to the receptor, resulting in the opening of a large pore and the following increase of potassium efflux^11,12^. Nigericin, another simulator of NLRP3 inflammasome, a microbial toxin derived from *Streptomyces hygroscopicus*, induces the potassium efflux through pannexin-1^13^. Activation of NLRP3 inflammasome by nigericin is independent of P2X7 receptor, because nigericin itself acts as a potassium ionophore. Celastrol blocked nigericin-induced production of caspase-1(p10) and cleaved IL-1β in macrophages (Supplemental Fig. 2A). Nigericin-induced IL-1β secretion was blocked by celastrol (Supplemental Fig. 2B). Nigericin-induced ASC oligomerization was also suppressed by celastrol in macrophages (Supplemental Fig. 2C,D). These suggest that the inhibitory effects of celastrol may not be dependent on P2X7 receptor but related to the process of potassium efflux.

**Figure 7:**
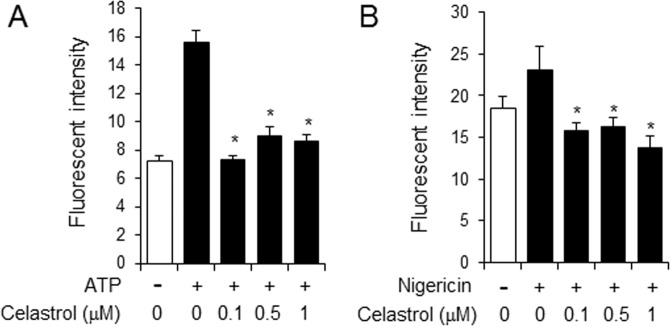
Celastrol suppresses the potassium efflux induced by ATP and nigericin in primary macrophages. Primary mouse macrophages were primed with LPS (100 ng/ml) for 4 hr. The cells were treated with celastrol for 1 hr and then stimulated with (**A**) ATP (5 mM) or (**B**) nigericin (10 μM) together with potassium binding benzofuran isophthalate (PBFI)-tetraammonium salt for 1 hr. Values are means ± SEM (n = 3). *Significantly different from ATP or nigericin alone, *p* < 0.05.

To investigate the inhibitory effect of celastrol on the potassium efflux induced by ATP or nigericin, we determined the level of intracellular potassium ion after stimulation of macrophages with ATP and nigericin using a potassium sensitive dye, potassium binding benzofuran isophthalate (PBFI)-tetraammonium salt. The level of the potassium efflux was increased upon ATP stimulation while celastrol suppressed ATP-induced potassium efflux in macrophages (Fig. 7A). Furthermore, nigericin-induced potassium efflux was also reduced by celastrol (Fig. 7B). These results indicate that celastrol suppressed ATP-induced activation of NLRP3 inflammasome by blocking the process of potassium efflux in macrophages.

## Discussion

Inflammation and immunity are considered important determinants of tumorigenesis from onset to cancer equilibrium during cancer development^14,15^. Once tumorigenesis is initiated by autonomous aberration of cells, the interaction between cancer cells and their microenvironment, particularly immune cells, is critical to govern tumor evasion. Much effort has been devoted to decoding the functions of the adaptive immune system in tumor immunity surveillance^15,16^, however, the contribution of innate immune pathways to this process is less understood. Among the effectors of the innate immune system, gonad-encoding pattern recognition receptors (PRRs) trigger inflammatory responses when detecting invading microorganisms or danger-associated molecules. Many PRRs, including members of the NOD-like receptor (NLR) and the HIN200 families, work by combining macromolecular complexes called inflammasomes^4^. This signaling scaffold induces innate immunity by activating protease caspase-1, which cleaves the immune and metabolic matrix^17,18^. In particular, it induces the production of pro-inflammatory cytokines, IL-1β and IL-18, resulting in inflammation and pyroptosis^19^. Guo *et al*. showed that IL-1β enhanced the growth and metastasis of tumor cells and the recruitment of myeloid cells to tumor tissue sites in breast cancer mouse models, suggesting that tumor microenvironment produces IL-1β to promote the metastatic potential of tumor cells^20^. Late-stage human melanoma cells constitutively secrete IL-1β through spontaneous activation of the NLRP3 inflammasome, promoting inflammatory environment, macrophage chemotaxis, and angiogenesis^21^. Our results demonstrate that IL-1β produced by macrophages stimulates the metastatic potential of melanoma cells, such as migration and invasion properties, showing the essential role of myeloid cells in the production of IL-1β in tumor microenvironment through activation of the NLRP3 inflammasome. Furthermore, inhibition of the NLRP3 inflammasome in myeloid cells by a pharmacological inhibitor such as celastrol resulted in the decreased metastatic potential of melanoma cells. These results suggest the importance of NLRP3 inflammasome in the tumor microenvironment in the regulation of tumor metastasis. Therefore, the results further provide a beneficial strategy for preventing tumor metastasis by regulating NLRP3 inflammasome in immune cells and thereby reducing IL-1β levels in tumor sites.

Interestingly, the NLRP3 inflammasome plays different roles in the tumorigenesis of melanoma in a stage-dependent manner^22^. Silencing ASC expression resulted in G1 cell cycle arrest, reduced cell viability, and downregulation of tumorigenesis in metastatic melanoma while silencing ASC expression in primary melanoma led to increased cell viability with enhanced tumorigenesis^22^. In addition, Drexler *et al*. suggested that ASC exhibited different impacts on tumorigenesis depending on the different cell types^23^. Mice had more tumors than control group when ASC was specifically deficient in keratinocytes, suggesting that ASC may be a tumor-suppressor in keratinocytes^23^. The expression level of ASC protein in human metastatic melanoma was lower than in primary melanoma and ASC protein expression was lost in human cutaneous squamous cell carcinoma^22,23^. In contrast, similar to our results showing the role of NLRP3 inflammasome in myeloid cells in enhancing the metastatic potential, mice with a specific deficiency of ASC in myeloid cells developed reduced skin cancer incidence, showing its pro-inflammatory role in myeloid cells^23^.

The influence of the NLRP3 inflammasome appears controversial in colitis-associated colon cancer. Unlike our results and those of Drexler *et al*., the NLRP3 inflammasome expressed in hematopoietic cells exerted a protective role against colitis-associated cancer as the increased tumor burden correlated with the downregulation of IL-1β expression at the tumor site^24^. Caspase-1 deficiency resulted in enhanced tumor formation in the azoxymethane and dextran sodium sulfate colitis-associated colorectal cancer mouse model through the regulation of colonic epithelial cell proliferation and apoptosis, but not through the regulation of inflammatory cells^25^.

The anti-inflammatory properties of celastrol have previously been reported. Celastrol inhibited lipopolysaccharides (LPS)-induced the activation of immune cells, mediated through the suppression of the transcription factors NF-κB and AP-1^26^. We previously reported that the inhibitory effects of celastrol on LPS-induced inflammation were mediated by blocking LPS binding to TLR4/MD2 complex in macrophages^27^. In this study, we show that celastrol suppressed the activation of NLRP3 inflammasome induced by ATP or nigericin. Celastrol inhibited the activation of NLRP3 inflammasome induced by another activator, uric acid crystals (Supplemental Fig. 3) although the inhibitory potency was weak. Interestingly, celastrol did not suppress the activation of other inflammasomes such as AIM2 nor NLRC4 inflammasome in primary mouse macrophages (Supplemental Fig. 1). Decrease of cell viability was observed by ATP treatment possibly due to increase of pyroptosis accompanied by NLRP3 inflammasome activation (Supplemental Fig. 4). However, treatment of celastrol in addition to ATP did not significantly enhance cell death (Supplemental Fig. 4). Furthermore, celastrol did not show any dose-dependent effect on cell viability (Supplemental Fig. 4). It is reported that inhibition of NLRP3 inflammasome by celastrol is possibly mediated through the down-regulation of reactive oxygen species production and NF-κB activation^28^. In addition, the induction of autophagy was suggested as the mechanism by which celastrol inhibits the NLRP3 inflammasome^29^. Our results demonstrate that blocking potassium efflux is a new mechanism for celastrol inhibiting the NLRP3 inflammasome in macrophages.

Several anti-cancer targets and mechanisms for celastrol have been studied in cancer cells. Celastrol-induced autophagy blocked gastric cancer growth^30^ and celastrol strongly downregulated the protein levels of Bcr-Abl in chronic myelogenous leukemia cells^31^. The anti-tumor effects of celastrol on MCF-7 cells were mediated through AMPK-induced p53-polo-like kinase 2 (PLK-2) pathways^32^. Celastrol suppressed the activation of STAT3/JNK 1/2/c-Src pathway in human hepatocellular carcinoma^33^. Celastrol is a proteasome inhibitor leading to enhanced apoptosis in prostate cancer models^34^. Our results propose the NLRP3 inflammasome as a new anti-cancer target of celastrol for the regulation of myeloid cell activation in tumor microenvironment. The inhibition of NLRP3 inflammasome by celastrol in macrophages results in the suppression of tumor metastatic potential. The results would provide a novel anti-cancer strategy using NLRP3 inflammasome inhibitors, such as celastrol to modulate the myeloid cells in tumor microenvironment and tumor metastatic potential by reducing the IL-1β level at tumor sites.

ATP abundant in tumor microenvironment stimulates the NLRP3 inflammasome in macrophages, releasing mature IL-1β to the tumor microenvironment to enhance the metastatic potential of cancer cells. Our results demonstrate that suppression of ATP-induced NLRP3 inflammasome activation and IL-1β secretion in tumor microenvironment, contributes to decreasing metastatic potential of cancer cells. Our results further suggest a possible therapeutic application of NLRP3 inflammasome inhibitors that would prevent the onset of NLRP3 inflammasome-mediated inflammatory and immune diseases.

## Materials and Methods

### Animals and cell culture

Animal care and the study protocols were carried out in accordance with the guidelines of the Institutional Animal Care and Use Committee (IACUC) of the Catholic University of Korea. The experimental protocols were approved by the IACUC of the Catholic University of Korea with a permission # 2012-5-001. C57BL/6 mice were purchased from Orient Bio (Seoul, Korea). NLRP3-knockout mice (B6N.129-Nlrp3tm3Hhf/J) and caspase-1 knockout mice (B6N.129S2-Casp1tm1Flv/J) were purchased from The Jackson Laboratory (Bar Harbor, ME, USA). The mice were acclimated under specific pathogen-free conditions in an animal facility for at least a week before the experiments. They were housed in a room controlled for temperature (23 ± 3 °C) and relative humidity (40–60%) and acclimated under specific pathogen-free conditions in the animal facility for at least a week before the experiments.

Bone marrow cells were isolated from C57BL/6 mice and differentiated to macrophage as described previously^35^. Bone marrow cells were cultured in in Dulbecco Modified Eagle Medium (DMEM) (Gibco, Hampton, NH, USA) containing 10% (v/v) heat-inactivated fetal bovine serum (Corning, NY, USA), 2 mM L-glutamine, 1 mM sodium pyruvate, 10 mM Hepes buffer and 20% L929 cell-conditioned medium for 6 days, and adherent cells were used as macrophages. Mouse melanoma cell line (B16F10, CRL-6475, American Type Culture Collection, Manassas, VA, USA) were cultured in DMEM containing 10% (v/v) heat-inactivated fetal bovine serum, 100 units/ml of penicillin and 100 μg/ml of streptomycin (Gibco). The cells were maintained at 37 °C in a 5% CO_2_/air environment.

For inflammasome activation study, primary mouse macrophages were primed with lipopolysaccharides (LPS) for 4 h. To examine the effect of celastrol on inflammasome with excluding the effect on LPS, LPS was removed by washing with phosphate-buffered saline (PBS) before treatment of celastrol. After cells were treated with celastrol for 1 h, cells were stimulated with inflammasome activators such as adenosine triphosphate (ATP), nigericin, monosodium uric acid crystals, poly dA:dT, or flagellin.

### Reagents

Purified LPS from *Escherichia coli* was obtained from List Biological Laboratory Inc. (Campbell, CA, USA) and dissolved in endotoxin-free water. Celastrol was purchased from Sigma-Aldrich (St. Louis, MO, USA) and dissolved in dimethyl sulfoxide (DMSO). ATP and flagellin were purchased from Invivogen (San Diego, CA, USA). Nigericin was purchased from Sigma-Aldrich.

### *In vitro* migration assay

Cellular migration assay was performed using Boyden transwell chamber (8-μm pore size, Becton Dickinson, Bedford, MA). Tumor cells (2 × 10^5^ cells/100 μl) were placed in the filter membrane. Conditioned medium (300 μl) obtained from primary mouse macrophages were placed in the lower chamber. After transwell chambers with cells were maintained at 37 °C for 24 h, cells on the upper membrane surface were completely removed by wiping with cotton swab and the migrant cells on the lower membrane surface were fixed with 4% paraformaldehyde and then stained with 0.5% crystal violet (Sigma-Aldrich). The membranes were examined microscopically and stained migrant cells were counted in at least 5 randomly selected fields.

### *In vitro* invasion assay

Cell invasion assay were performed using Boyden transwell chamber (8-μm pore size, Becton Dickinson). Matrigel (Becton Dickinson) diluted with serum-free DMEM was placed in the upper chamber of filter membrane, and incubated at 37 °C for 4 h. A total of 2 × 10^5^ tumor cells were seeded with serum-free DMEM in the upper chamber. Conditioned medium (300 μl) obtained from bone-marrow derived macrophages were placed in the lower chamber. After transwell chambers with cells were maintained at 37 °C for 24 h, cells on the upper membrane surface were completely removed by wiping with cotton swab and the migrant cells on the lower membrane surface were fixed with 4% paraformaldehyde and then stained with 0.5% crystal violet (Sigma-Aldrich). The membranes were examined microscopically and stained migrant cells were counted in at least 5 randomly selected fields.

### Immunoblotting analysis

This was performed as described previously^36^. The antibody to detect pro-caspase-1 (45 kDa) and caspase-1(p10) (10 kDa) was obtained from Santa Cruz Biotechnology (sc-514, Dallas, TX, USA). An antibody to detect pro-IL-1β (31 kDa) and mature IL-1β (17 kDa) was from R&D systems (Minneapolis, MN, USA).

### ELISA

The levels of IL-1β in culture media were determined using enzyme-linked immunosorbent assay (ELISA) kits (R&D Systems, Minneapolis, MN, USA). The concentration ranges for the standard curves is from 12.5 to 1000 pg/mL and the minimum detectable dose ranged from 0.46 to 4.8 pg/mL concentration ranges for the standard curves.

### Determination of ASC oligomerization

This was performed as described previously^37^. Cell pellet fractions were immunoblotted with anti-ASC antibody (sc-22514-12, Santa Cruz Biotechnology).

### Confocal imaging analysis

This was performed as described previously^38^. Briefly, after LPS-primed primary macrophages were treated with celastrol for 1 h, cells were stimulated with ATP and nigericin. Cells were fixed and incubated with anti-ASC antibody (sc-22514-12, Santa Cruz Biotechnology) and DAPI (4′, 6-diamidino-2-phenylindole). Cells were further incubated with FITC-conjugated anti-rabbit IgG antibody (Sigma-Aldrich). The samples were examined with an LSM710 confocal laser scanning microscope (Carl Zeiss, Oberkochen, Germany) equipped with 40x objectives. Images were obtained with ZEN2011 software (Carl Zeiss).

### Determination of extracelluar potassium concentration

Primary mouse macrophages were grown in 96-well plates until 80–90% confluency. After cells were pre-treated with celastrol for 1 h, cells were stimulated with ATP and nigericin together with potassium binding benzofuran isophthalate (PBFI)-tetraammonium salt (Molecular Probes, Inc., Eugene, OR, USA) for 1 h. Samples were read with fluorescence plate reader (SpectraMax M5, Molecular Devices, Sunnyvale, CA, USA) at excitation 340 nm and emission 515 nm.

### Statistical analysis

Data are expressed as means ± SEM (n = 3–5). Comparisons of data between groups were performed by one-way analysis of variance (ANOVA) followed by Duncan’s multiple range test. Values of *p* < 0.05 were considered significant.

## Supplementary information

Supplementary Information
