# Supplementary material for: Inhibition of NLRP3 inflammasome in tumor microenvironment leads to suppression of metastatic potential of cancer cells
Source: Sci Rep. 2019 Aug 22;9:12277. doi: 10.1038/s41598-019-48794-x (PMC6706417; doi:10.1038/s41598-019-48794-x)

Supplementary information

Inhibition of NLRP3 inflammasome in tumor microenvironment leads to suppression of metastatic potential of cancer cells

Hye Eun Lee[1,*], Jin Young Lee[1,*], Gabsik Yang[1], Han Chang Kang[1], Yong-Yeon Cho[1], and Hye Suk Lee[1], and Joo Young Lee[1‡]

[1]BK21 PLUS Team, College of Pharmacy, The Catholic University of Korea, Bucheon, Republic of Korea, 14662

[‡]Corresponding author: Joo Young Lee, College of Pharmacy, The Catholic University of Korea, Bucheon, Korea, 14662. Tel: 82-2-2164-4095. Fax: 82-2-2164-4059. E-mail: **joolee@catholic.ac.kr**.

*, equally contributed.

Running title: Suppression of metastatic potential by NLRP3 inflammasome inhibition in macrophages

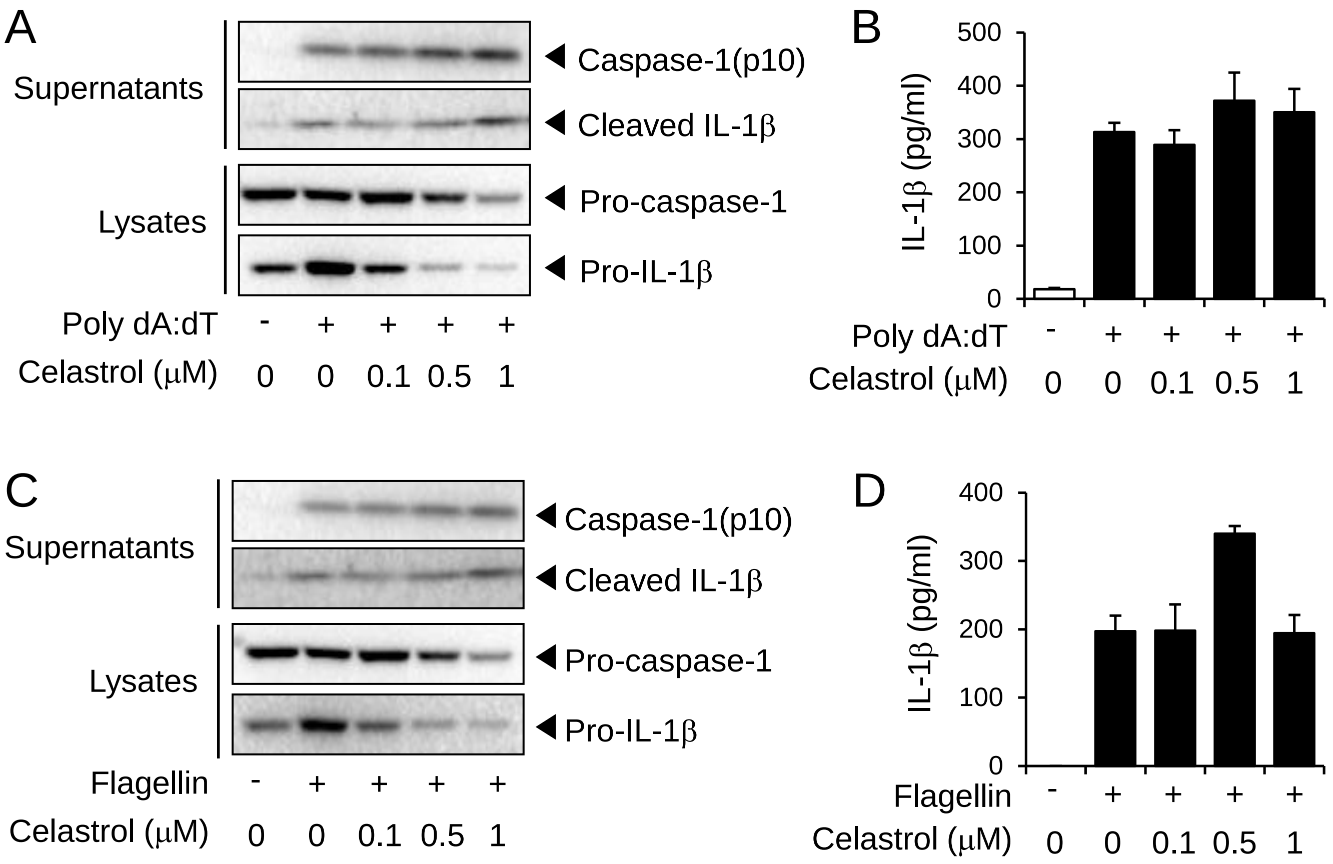

Supplemental Figure 1. Celastrol does not suppress the activation of AIM2 and NLRC4 inflammasome in primary macrophages. Bone marrow-derived macrophages were primed with LPS (500 ng/ml) for 4 hr. Then, the cells were treated with celastrol for 1 hr and further stimulated with (A, B) poly dA:dT (1 μg) or (C, D) flagellin (10 μg) for 6 hr. A, C. Cell culture supernatants and cell lysates were immunoblotted for pro-caspase-1, caspase-1(p10), pro-IL-1β, and IL-1β. B, D. Cell culture supernatants were analyzed for secreted IL-1β using ELISA. Values represent the means ± SEM (n=3-5).

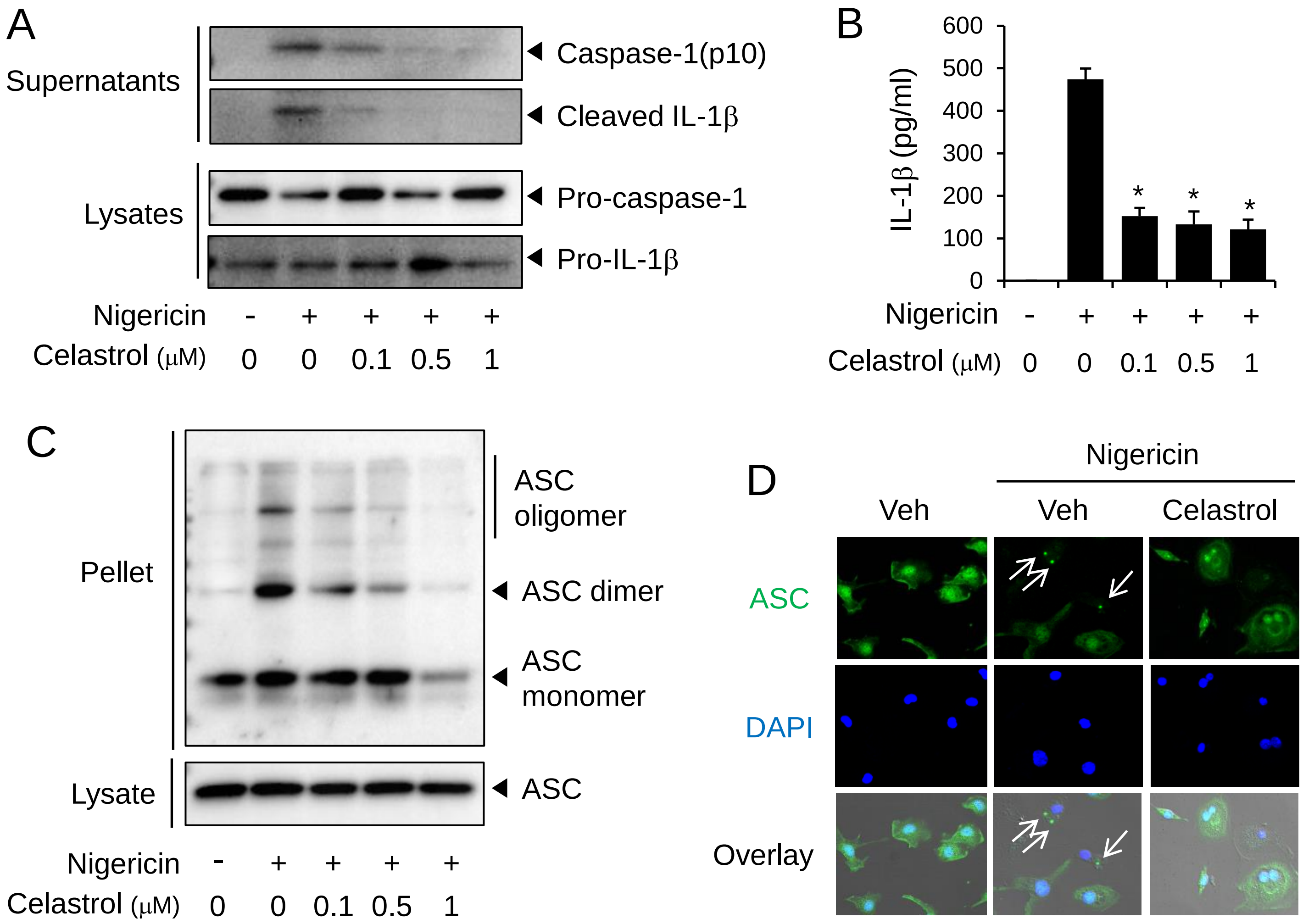

Supplemental Figure 2. Celastrol suppresses NLRP3 inflammasome activation induced by nigericin in primary macrophages. Bone marrow-derived macrophages were primed with LPS (100 ng/ml) for 4 hr. The cells were treated with celastrol for 1 hr and then stimulated with nigericin (10 μM) for (A) 1 hr or (B) 16 hr. (A) The cell culture supernatants and cell lysates were immunoblotted for pro-caspase-1, caspase-1(p10), pro-IL-1β, and IL-1β. (B) The cell culture supernatants were analyzed for secreted IL-1β using ELISA. Values represent the means ± SEM (n=3). *, significantly different from nigericin alone, p <0.05. (C) The cell lysates were immunoblotted for ASC. (D) The cells were stained for ASC (green), and the nuclei were stained with 4',6-diamidino-2-phenylindole (DAPI; blue). The arrows indicate ASC speckles. The data are representative of three independent experiments. For immunoblotting results, the cropped blots from full length gels were presented.

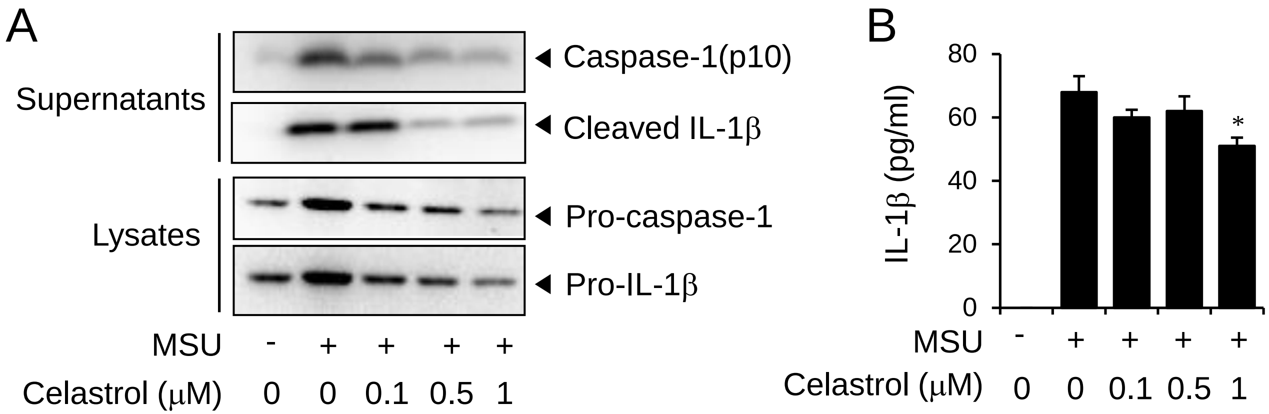

Supplemental Figure 3. Celastrol suppresses NLRP3 inflammasome activation induced by uric acid crystals in primary macrophages. Bone marrow-derived macrophages were primed with LPS (500 ng/ml) for 4 hr. The cells were treated with celastrol for 1 hr and then stimulated with monosodium uric acid crystals (MSU, 10 μg/ml) for 6 hr. (A) The cell culture supernatants and cell lysates were immunoblotted for pro-caspase-1, caspase-1(p10), pro-IL-1β, and IL-1β. (B) The cell culture supernatants were analyzed for secreted IL-1β using ELISA. Values represent the means ± SEM (n=3). *, significantly different from MSU crystals alone, p <0.05.

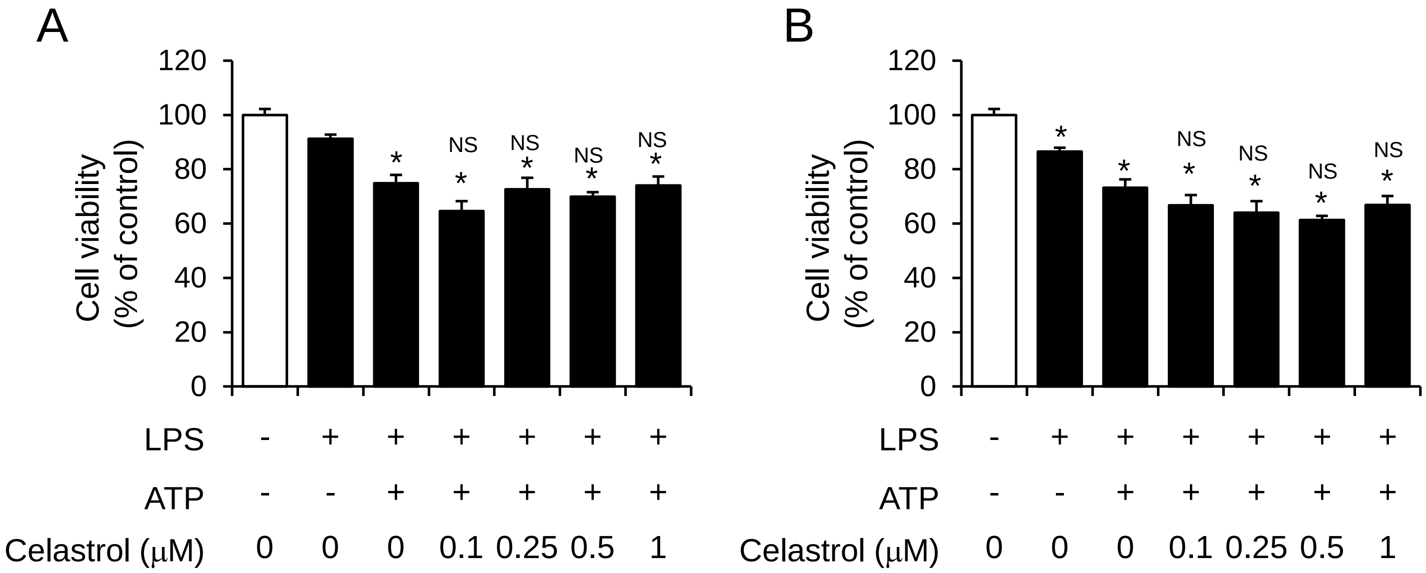

Supplemental Figure 4. Cell viability. Bone marrow-derived macrophages were primed with LPS (A: 100 ng/ml, B: 500 ng/ml) for 4 hr. The cells were treated with celastrol for 1 hr and then stimulated with ATP for 2 hr. Cell viability was determined by MTT assay. Values are expressed as the % of control (white bar). Values represent the means ± SEM (n=6). Comparisons of data between groups were performed by one-way analysis of variance (ANOVA) followed by Duncan's multiple range test. *, significantly different from vehicle alone, p <0.05. ns, not significant from LPS+ATP.

Lee et al. Figure 5: Full gel pictures

Figure 5B
Caspase-1(p10)

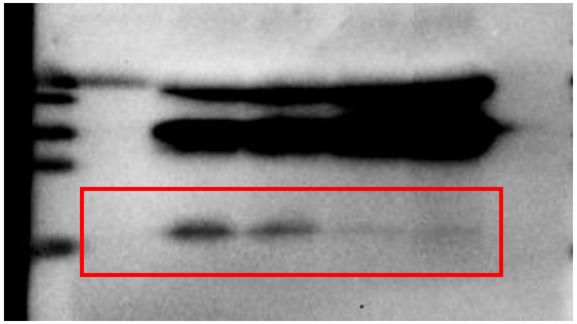

Figure 5B
Pro-caspase-1

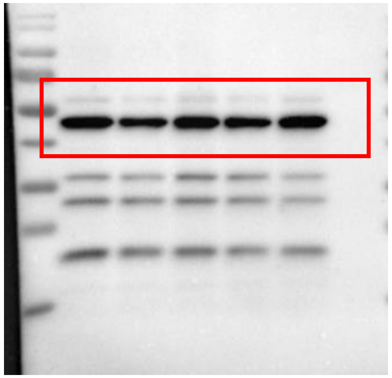

Figure 5D
ASC oligomer

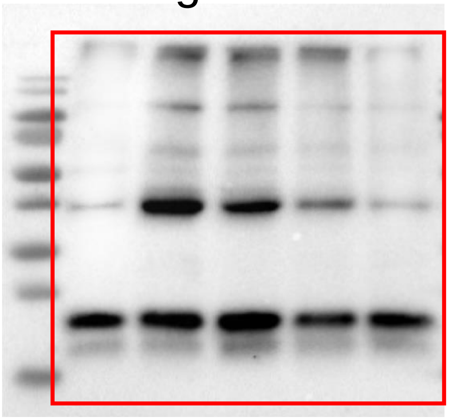

Figure 5B
Cleaved IL-1β

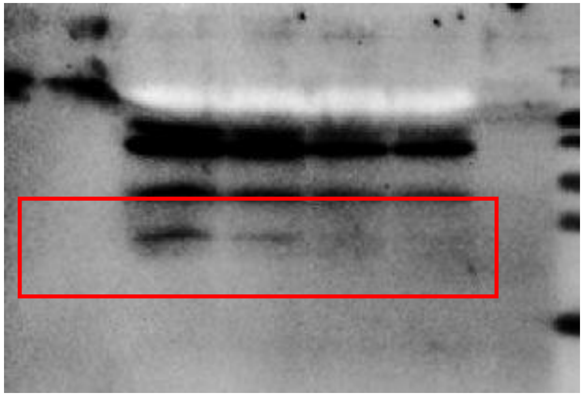

Figure 5B
Pro-IL-1β

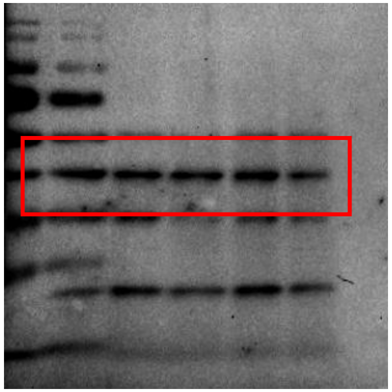

Figure 5D
ASC

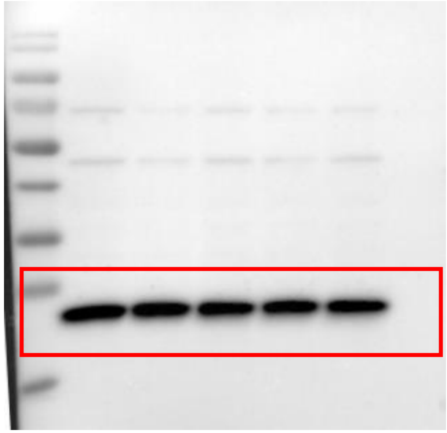

Lee et al. Supplemental Figure 2: Full gel pictures

S Figure 2A
Caspase-1(p10)

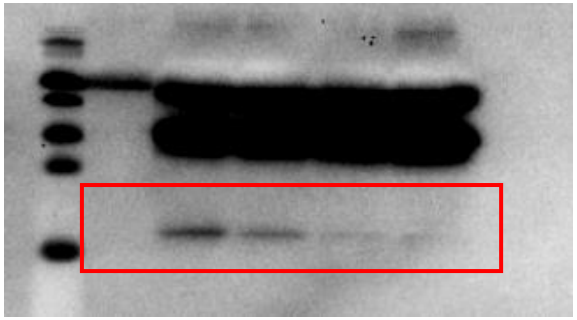

S Figure 2A
Pro-caspase-1

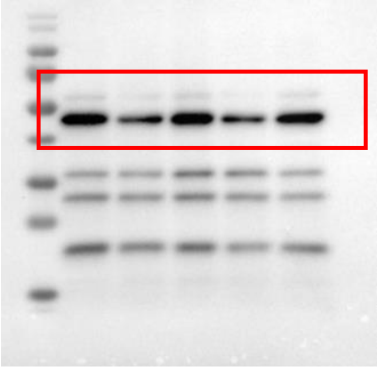

S Figure 2C
ASC oligomer

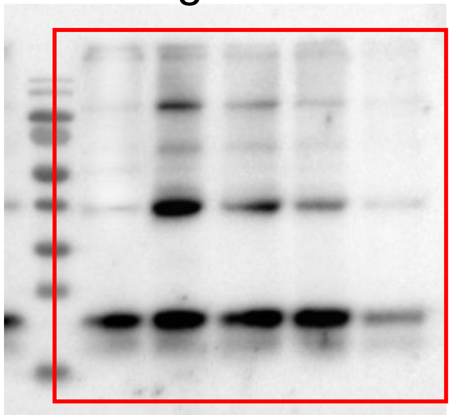

S Figure 2A
Cleaved IL-1β

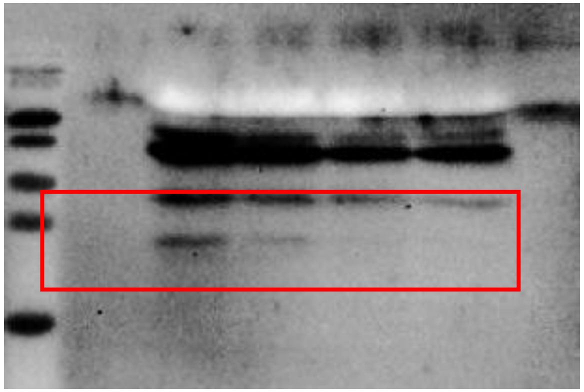

S Figure 2A
Pro-IL-1β

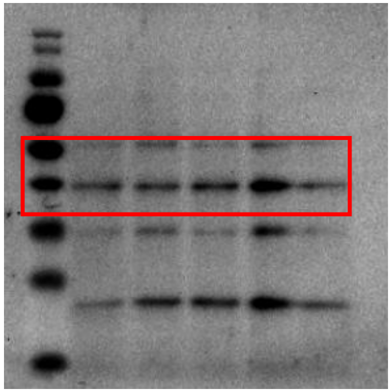

S Figure 2C
ASC

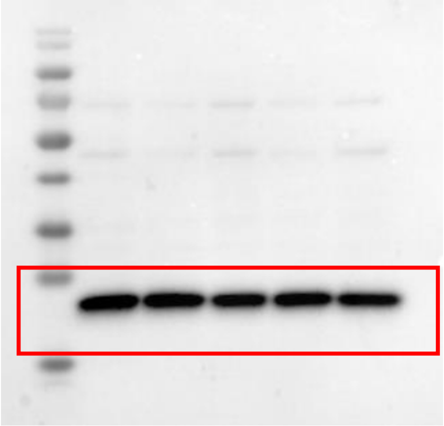

Supplement: Supplementary file 1 — Supplementary Information [file 41598_2019_48794_MOESM1_ESM.pdf]
